# Shared Neural Substrates of Emotionally Enhanced Perceptual and Mnemonic Vividness

**DOI:** 10.3389/fnbeh.2013.00040

**Published:** 2013-05-06

**Authors:** Rebecca M. Todd, Taylor W. Schmitz, Josh Susskind, Adam K. Anderson

**Affiliations:** ^1^Department of Psychology, University of TorontoToronto, ON, Canada; ^2^MRC Cognition and Brain Sciences Unit, University of CambridgeCambridge, UK; ^3^Institute for Telecommunications and Information Technology, University of California San DiegoSan Diego, CA, USA

**Keywords:** memory, emotion, amygdala, visual cortex, emotional salience, affect-biased attention, emotionally enhanced memory, fMRI

## Abstract

It is well-known that emotionally salient events are remembered more vividly than mundane ones. Our recent research has demonstrated that such memory vividness (Mviv) is due in part to the subjective experience of emotional events as more perceptually vivid, an effect we call emotionally enhanced vividness (EEV). The present study built on previously reported research in which fMRI data were collected while participants rated relative levels of visual noise overlaid on emotionally salient and neutral images. Ratings of greater EEV were associated with greater activation in the amygdala and visual cortex. In the present study, we measured BOLD activation that predicted recognition Mviv for these same images 1 week later. Results showed that, after controlling for differences between scenes in low-level objective features, hippocampus activation uniquely predicted subsequent Mviv. In contrast, amygdala and visual cortex regions that were sensitive to EEV were also modulated by subsequent ratings of Mviv. These findings suggest shared neural substrates for the influence of emotional salience on perceptual and mnemonic vividness, with amygdala and visual cortex activation at encoding contributing to the experience of both perception and subsequent memory.

## Introduction

It is well-known that emotionally important events such as the birth of a child or September 11 hold a special place in memory (e.g., Brown and Kulik, 1977). There is substantial empirical evidence that emotionally salient stimuli or events are more likely to be remembered than mundane ones (Ochsner, 2000) and are remembered more vividly (Sharot et al., 2004, 2007; Kensinger et al., 2011).

There is also abundant evidence that emotional salience influences the initial perception of events. Emotions are associated with the mutually enhancing effects of sympathetic arousal (Anderson et al., 2006b) and increased attention (Pessoa et al., 2002; Anderson et al., 2003; Talmi et al., 2008), which results in facilitated encoding of emotional events (Anderson and Phelps, 2001; Anderson, 2005; De Martino et al., 2009; Lim et al., 2009). Emotionally important stimuli are also more easily detected and identified than neutral stimuli when attentional load is high (Soares and Ohman, 1993; Anderson, 2005), or stimuli are presented at the threshold of awareness (Nielsen and Sarason, 1981). At the neural level, human fMRI studies have shown that viewing emotionally salient images, such as scenes of mutilation or erotica, is associated with enhanced engagement of regions of visual cortex (Lang et al., 1998; Bradley et al., 2003; Vuilleumier et al., 2004; Sabatinelli et al., 2005; Padmala and Pessoa, 2008).

We have previously demonstrated that emotion-enhanced memory vividness (Mviv) in part reflects a subjective richness of perceptual experience, or emotionally enhanced vividness (EEV) (Todd et al., 2012). Using a magnitude estimation task in which low levels of visual noise were overlaid on neutral and emotionally salient scenes, we demonstrated that when objective levels of noise were equated, arousing images were experientially perceived as more perceptually vivid than neutral images (Todd et al., 2012). That is, participants subjectively perceived emotionally arousing images as containing greater signal (the underlying image) relative to overlaid noise. fMRI data revealed that, after controlling for low-level features of stimuli such as contrast, color, and scene complexity, EEV ratings were positively correlated with activity in left amygdala and lateral occipital cortex (LOC). Statistical mediation analyses suggested that amygdala activation accounted for the influence of visual cortex activation on EEV (Todd et al., 2012).

We also reported behavioral data indicating that EEV predicted subsequent Mviv, measured both by cued recall 45 min after encoding and recognition memory 1 week later (Todd et al., 2012). Previous studies have shown that enhanced memory for arousing images is associated with greater amygdala and visual cortex activation during encoding (Hamann et al., 1999; Canli et al., 2000; Kensinger et al., 2007b). Since our results showed that amygdala and visual cortex were modulated by EEV, an outstanding question concerned whether activity in these same regions predicts subsequent Mviv.

**Figure 1:**
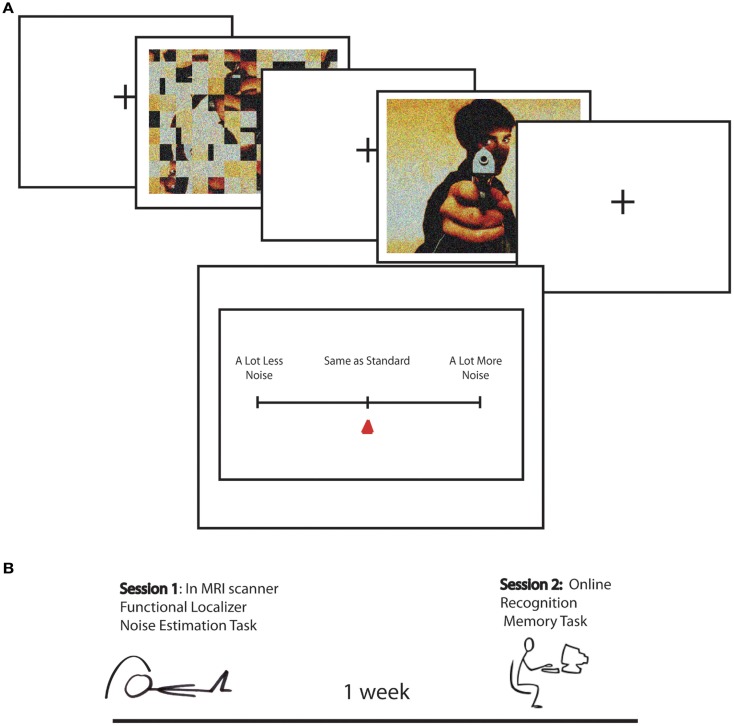
**(A)** Task design for Noise Estimation fMRI experiment. A standard, created by phase scrambling the comparison image, was overlaid with 15% noise. The standard was followed by the image overlaid with 10, 15, or 20% noise. Following image offset, participants moved a cursor on a scale to indicated NE for the image relative to the standard from “a lot less noise” to “same as standard” to “a lot more noise.” **(B)** Schematic of the study timeline over two sessions. In Session 1 participants performed the Noise Estimation task in the scanner. One week later they performed an online recognition memory task in which they were asked if images were old or new, and if they were old how vividly they were remembered on a scale from vague to detailed.

The present study built on a previously reported study (Todd et al., 2012), in which to measure EEV we adapted a magnitude estimation paradigm designed to estimate human subjective estimates of graded magnitudes of sensory stimuli (Stevens, 1957). In each version of the noise estimation (NE) task, emotionally arousing (positive and negative) and neutral scenes were overlaid with sparse “visual noise,” or randomly distributed pixels. Participants were asked to estimate the relative degree of “noisiness” of each picture in comparison to a standard image, which was created from scrambled versions of the scenes, matched in global luminance and contrast, overlaid with a varying levels of sparse noise (Figure 1). This provided a direct behavioral index of whether greater emotional salience results in greater perceptual vividness.

The goal of the present study was to investigate the link between the subjective experience of perceptual vividness and mnemonic vividness. Here we examined BOLD activation predicting Mviv ratings collected from an online recognition memory task 1 week after participants were scanned during the NE procedure in relation to activation evoked by EEV (for a timeline of the procedure, see Figure 1B). We predicted that amygdala and LOC activation would predict Mviv, along with additional brain regions, such as the hippocampus, also implicated in episodic memory (Lepage et al., 1998; Eldridge et al., 2005).

## Materials and Methods

### fMRI study

#### Participants

Eighteen healthy young adult volunteers from Queen’s University (18–30 years) with normal or corrected to normal vision and no history of psychiatric disorders participated in this study. Two participants were removed from subsequent fMRI analysis: one had excessive movement and one misunderstood the task, leaving 16 participants (10 female).

#### Localizer task

In order to localize category-selective regions of visual cortex, we used a block design task that alternated blocks of line-drawings of objects and scrambled line-drawings to localize object-selective regions of the LOC. The task also included blocks of faces and houses to localize face and place selective regions of visual cortex respectively. Blocks alternated randomly to minimize category predictability and each image category was presented six times. Each 20 s block contained five images presented for 4000 ms each. In each block one of the images was repeated. To ensure that they were attending to the images presented onscreen, participants were asked to push a button with the index finger each time an image appeared if the image was not the same as the one immediately preceding it, and to press a second button with the middle finger if the image was identical to the one immediately preceding it. Because the behavioral task served merely as a vigilance task to maintain attention and maximize BOLD activation, behavioral results were not analyzed.

### Noise estimation task

#### Materials

Twenty-five negative photos and 25 positive photos were taken from the International Affective Picture System (IAPS). Twenty-five neutral photos were retrieved from the internet as well as the IAPS. A separate set of participants rated all pictures to be equal in brightness, contrast, and visual complexity, *F*s(2, 72) < 0.1. Positive, negative, and neutral images were selected to be equivalent on basic low-level image statistics, equated in log luminance, *F*(2, 72) < 1, and RMS contrast, *F*(2, 72) < 1. One of three levels of Gaussian all-color noise (15%) was superimposed over each image using adobe Photoshop 7.0.

To minimize variance associated with differences in luminance and contrast across images, a standard was created to match each corresponding comparison image (Figure 1). Standards were created by phase scrambling each image and adding noise. Both standards and images subtended a visual angle of 13° × 9.5°. The level of noise on the pictures was held constant (15%) and noise level was varied in the standards (10, 15, 20%). Thus, we could measure BOLD responses related to estimations of perceptual and mnemonic vividness across images that did not vary in objective levels of noise.

Trials were presented in five separate runs of 30 trials. In order to reduce time spent into the scanner in this slow event-related design, each image was presented twice at two out of three levels of standard noise for a total of 150 trials (50 negative arousing, 50 positive arousing, and 50 neutral). Thus, each image was seen twice at 15% noise paired with a standard at two out of three possible noise levels (10, 15, and 20%). In each 12-s trial a standard was presented for 1500 ms, followed by a 500 ms ISI, followed by the picture which was presented for 1500 ms. Both standards and images subtended a visual angle of 13° × 9.5°. After a randomly jittered interval of 1500, 2000, or 2500 ms, a response meter appeared for 4000 ms, followed by a randomly jittered ITI of 2000, 2500, or 3000 ms. Participants clicked a button with the index finger to move the cursor to the left (less noisy than standard) and with the middle finger to move the cursor to the right (more noisy than standard), and a third button with the middle finger to indicate that the cursor was placed at the desired degree of noisiness. Fifty null trials (1/3), consisting of 12 s of fixation, were included at randomized intervals. After the scan participants were asked to log on to a website 1 week later.

#### Memory task

One week after performing the NE task, participants were instructed to log on to a website to complete a surprise memory task exactly 1 week after performing the NE task. An additional set of images, 12 positive, 12 neutral, and 12 negative, were matched with the Session 1 images for arousal, scene content, contrast, and luminance. Participants were shown the original 75 images and the 36 new images. After rating an image as new, familiar, or recollected, participants rated the vividness of the memory for recognized items on a scale of 1–7 (0 = new image), ranging from vague to detailed.

#### fMRI acquisition

Imaging data were collected with a 3T Siemens scanner using a 12-channel head coil. Both the localizer and experimental tasks were programed in E-prime Version 1.2 (Psychology Software Tools, Pittsburgh, PA, USA). For each subject, a three-dimensional magnetization prepared rapid-acquisition gradient-echo pulse sequence (MPRAGE) was used to acquire a high-resolution T1-weighted structural volume: repetition time (TR) = 1760 ms; echo time (TE) = 2.2 ms; FOV = 256 × 256; slice thickness = 1mm; 176 slices; total acquisition time = 7:32 min.

T2* weighted gradient-echo echo planar images were collected for two short field mapping series to correct for EPI distortion due to inhomogeneities in the magnetic field. Parameters for the field mapping series were: TR = 793 ms; TE1 = 5.19 ms, and TE2 = 7.65 ms; flip angle = 60; FOV = 211 mm. Thirty-five slices were acquired with a voxel size of 3.3 mm × 3.3 mm × 3.5 mm. EPI parameters for the two functional tasks were: TR = 2000 ms; TE = 25 ms; flip angle = 78°; FOV = 211mm. Thirty-five slices were acquired with a voxel size of 3.3 mm × 3.3 mm × 3.5 mm.

#### Preprocessing

Data were analyzed with Statistical Parametric Mapping software (SPM8, University College London, London, UK; http://www.fil.ion.ucl.ac.uk/spm/sofware/spm8). Slice timing correction of reconstructed images was performed after removing the first five time points from each functional run. Field maps were unwarped with the EPI time series and time series were realigned for motion and field distortion correction. T1-weighted structural images were co-registered to the EPI images, and then bias corrected and segmented using MNI template tissue probability maps. EPI images were then co-registered to the normalized segmented anatomical images. Finally, time series data were smoothed with a 6 mm full-width half maximum Gaussian kernel.

#### First-level statistical models

For each subject, first-level general linear models were applied to localizer data and data from the NE task. For the localizer data, boxcar stimulus functions were convolved with the canonical hemodynamic response function (HDR). Our goal was to compare regions correlated trial by trial with Mviv ratings with previously patterns of parametric modulation by emotionally enhanced perceptual vividness. We thus examined parametric modulation of the BOLD response by both perceptual and mnemonic vividness ratings for each trial across all stimulus categories (positive, negative, and neutral). For the experimental data, a delta function regressor was modeled for image onset and convolved with the canonical HRF for each trial in each analysis. For each subject, we created a first-level statistical parametric model (SPM). To capture variance due to low-level features of the images which could influence our results, we first entered four separate regressors modeling scene complexity, hue, contrast, and mean visual saliency (a measure of a combination of features that allow a part of an image to stand out from its surround) (Itti and Koch, 2001). We next entered a regressor for Mviv to interrogate regions parametrically modulated by mnemonic vividness. An additional regressor was added for perceptual vividness, or inverse NE (NE^−1^) (for details, see Todd et al., 2012) to further interrogate effects of Mviv relative to NE^−1^. Full results of group level analysis reported below can be found in Tables 1 and 2.

**Table 1:** **Regions parametrically modulated by Mviv > Pviv**.

Brain region	*x*	*y*	*z*	Voxels	*t*	*p*
L lingual-parahippocampal gyrus-hippocampus/BA 37	−27	−52	−5	70	5.01	<0.001
R inf orbital gyrus/BA 47	−30	−29	−20	16	3.87	<0.001
L middle occipital gyrus/BA 18–19	−30	−91	−19	43	3.79	<0.001
R fusiform-parahippocampal gyrus/BA 37	−30	−40	−11	23	3.46	<0.001

**Table 2:** **Regions parametrically modulated by Pviv > Mviv**.

Brain region	*x*	*y*	*z*	Voxels	*t*	*p*
L insula/BA 48	−36	−19	−22	37	4.74	<0.001
L sup temporal gyrus/BA 48	−51	−13	−2	143	4.47	<0.001
R sup temporal gyrus-Heschl gyrus/BA 48	−51	−22	−1	35	3.29	<0.001
R sup temporal pole/BA 38	−54	−8	−11	13	2.87	<0.002

*xyz = MNI coordinates. Cluster size as thresholded at p < 0.005, uncorrected*.

#### Second-level statistical models

For the localizer task, *T* contrast files for each condition (object drawings, scrambled objects, places, and faces) from each individual were entered into a one-way ANOVA, with condition as the single factor. Contrasts for (objects > scrambled objects) were used to specify category-selective activation in the LOC. An additional contrast was used to specify place-sensitive regions of parahippocampal cortex (houses > faces). Functionally defined masks were created using 10 mm spheres around maxima activations in the group maps (51, −76, 1 and −45, −79, 1) thresholded at *p* < 0.05 (FWE). Anatomical masks for right and left amygdala and right and left hippocampus were created from automated anatomical labeling (AAL) (Tzourio-Mazoyer et al., 2002) templates based on a spatially normalized high-resolution T1 single-subject data set using the MarsBaR toolbox (Brett et al., 2002). Together, these ROIs were used for small volume correction for the NE task.

To orthogonalize their comparison, contrast files for Mviv and NE^−1^ from a single first-level model were used. To enable contrasts between activation enhanced by each parametric modulator, contrast files were entered into a one-way repeated measures ANOVA (independence not assumed) at the group level. Initial SPMs were thresholded at a height threshold of *p* < 0.005, uncorrected, with a cluster extent threshold of 10 voxels (for rationale, see Lieberman and Cunningham, 2009). Primary results for hypothesized regions were corrected for family-wise error. Functionally and anatomically defined ROIs were used as masks for small volume correction based on *a priori* hypotheses. Voxels surviving a family-wise error corrected *p* < 0.05 were deemed statistically significant.

## Results

### Behavioral results

#### Noise estimation task

A two factor repeated measures ANOVA was performed on NE with standard noise (3) and emotion (3) as factors. Results showed a main effect of standard noise, *F*(2, 28) = 28.42, *p* < 0.001. A significant linear contrast, *F*(1, 14) = 32.01, *p* < 0.001, revealed noise ratings of the images to be relatively lower relative to the standard as standard noise levels got higher, again indicating that participants were accurate at gaging relative noise even when noise level was manipulated in the standard. There was also a main effect of emotion, *F*(2, 28) = 10.32, *p* < 0.001, with arousing images rated as less noisy – or more perceptually vivid – than neutral images, *F*(1, 14) = 12.50, *p* = 0.003. A modest standard noise × emotion interaction, *F*(4, 56) = 4.26, *p* < 0.05, revealed differences between positive and neutral images to be greater at the two lower levels of standard noise. Contrasts revealed that, as in previous studies both positive and negative images were rated as less noisy, or more vivid, than neutral images, *p*s < 0.05; unlike in previous studies, negative images were also rated as more vivid than positive images, *p* < 0.05.

#### Memory

A one-way ANOVA was performed on Mviv scores with emotion as a repeated measure. There was a main effect of emotion category, *F*(2, 28) = 9.33, *p* = 0.001. Contrasts revealed that negative images were remembered more vividly than neutral or positive images, *F*(1, 14) = 20.82, *p* = 0.001. There was no effect of emotion category on memory accuracy.

### fMRI results

#### Perceptual and mnemonic vividness

**Figure 2:**
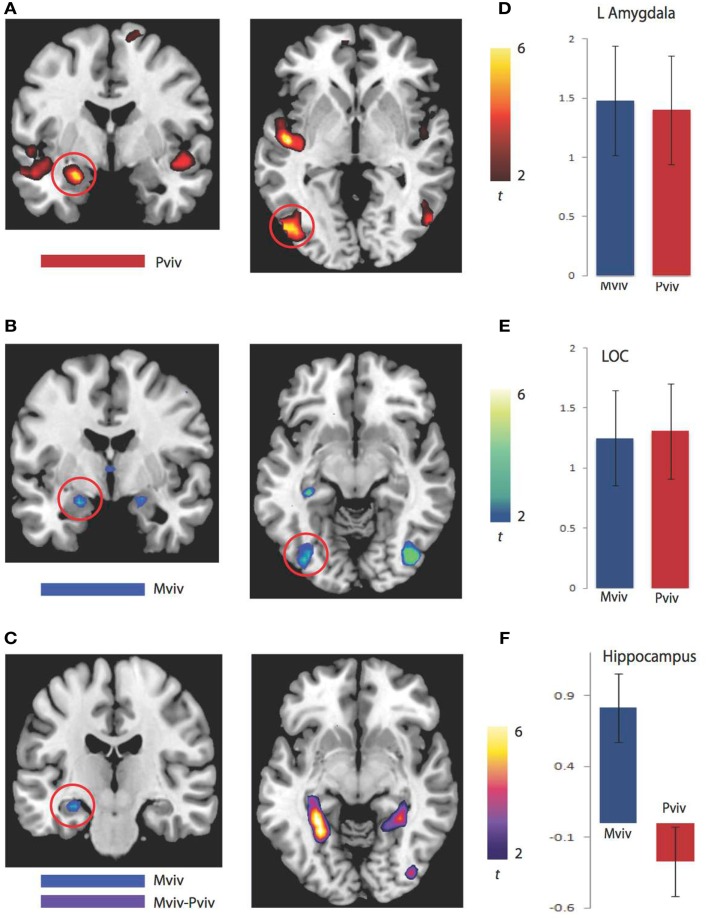
**BOLD correlates of perceptual and memory vividness**. **(A)** Activation maps for regions parametrically modulated by perceptual vividness. **(B)** Activation maps for regions parametrically modulated by memory vividness. **(C)** Activation maps showing hippocampal region activated by Mviv and regions activated by the contrast Mviv > Pviv. **(D)** Contrast estimates for Pviv and Mviv for left amygdala. **(E)** Contrast estimates for Pviv and Mviv for left LOC. **(F)** Contrast estimates for Mviv and Pviv for left hippocampal region activated by Mviv.

As previously reported, higher levels of NE^−1^ were associated with more activation in the left amygdala (*xyz* = −24, −4, −20) and left LOC (*xyz* = −51, −76, −2). Regions where greater activation was associated with higher levels of Mviv similarly included the left amygdala (−21, −4, −17; *t*_1, 28_ = 3.21, FWE *p* = 0.02, svc) and left LOC (−39, −79, −5; *t*_1, 28_ = 3.23, FWE *p* = 0.04, svc). To more closely examine the relation between regions modulated by memory and perceptual vividness, the contrast for Mviv was masked inclusively by activation for NE^−1^at a threshold of *p* = 0.005, uncorrected. All voxels activated by memory in the left amygdala and LOC fell within regions that were activated by perceptual vividness. As Figures 2D,E demonstrates, Mviv shows a highly similar pattern of activation to NE^−1^ in the left amygdala and left LOC. These voxels were also activated significantly in both conditions when analyzed separately.

To reveal regions uniquely recruited by Mviv, comparisons between Mviv and NE^−1^ revealed that a region of left hippocampus (−30, −37, −8; *t*_1, 28_ = 3.36, FWE *p* = 0.05, svc) and a place-sensitive region of left parahippocampal gyrus (−27, −52, −5; *t*_1, 14_ = 5.33, FWE *p* = 0.05, svc) were activated more for Mviv than NE^−1^ (Figure 2). Thus, consistent with our previously reported behavioral evidence of partial independence between EEV and Mviv, only a subset of regions activated by Mviv also reflected EEV.

## Discussion

Our results showed that, after controlling for differences between scenes in low-level objective features, amygdala and visual cortex regions that were sensitive to EEV were also modulated by ratings of subsequent Mviv. In contrast, hippocampus activation uniquely predicted Mviv. These findings suggest shared neural substrates for the influence of emotional salience on perceptual and mnemonic vividness, with amygdala and visual cortex regions associated with EEV at encoding contributing to the experience of emotionally enhanced memory.

Emotionally salient images (both positive and negative) are more likely to be remembered than neutral images (Ochsner, 2000). In particular, emotionally salient images have been linked to greater memory for the central, emotionally salient, or goal-relevant elements of a scene (Kensinger et al., 2007a; Levine and Edelstein, 2009). Such findings have been associated with greater amygdala activation (Hamann et al., 1999; Canli et al., 2000; Kensinger et al., 2007b) as well as high-order visual cortex activation at encoding (Kensinger et al., 2007b; Talmi et al., 2008), and suggest that perceptual processing of emotionally important events may be related to heightened memory (Kensinger et al., 2007b). Here we provide novel evidence for a direct mapping between emotion-enhanced perceptual vividness and mnemonic vividness.

Yet this finding is in contrast to a levels of processing framework (Craik and Lockhart, 1972), where it is categorization at a deeper conceptual level rather than shallower perceptual level that yields enhanced memory. Rather, our data suggest that emotional salience is associated with a unique behavioral and neural expression of memory. High levels of emotional salience, as tagged by the amygdala, may heighten the binding between objects, their meaning, and emotional significance (Nashiro and Mather, 2011), yielding a unique subjective vividness to both perception and memory.

In previous studies we found no differences between noise estimation ratings for positive and negative images (Todd et al., 2012). In the present study, although both positive and negative images were rated as less noisy than neutral images, negative images were further rated as less noisy, or more perceptually vivid, than positive images. This pattern of stronger results for negative stimuli extended to the memory findings in which negative images were more vividly remembered 1 week later than positive or negative images. Because of our parametric analysis across all trial types, we cannot report the extent to which image valence may have contributed to the pattern of results found here. Previous studies looking at emotionally enhanced memory have found amygdala activation at encoding to be associated with subsequent memory for both negative and positive stimuli (Hamann, 2001).

As previously reported, amygdala activation accounted statistically for the relation between LOC activation and EEV at the time of encoding (Todd et al., 2012). Although it is not possible to infer directionality from a mediation analysis, one hypothesis is that the enhanced visual cortex activation associated with EEV is amygdala-driven, and that a similar modulation of visual cortex by the amygdala produces subsequent Mviv. This hypothesis is consistent with a body of non-human animal research finding that emotional memory is characterized by arousal-enhanced noradrenergic activity in the basolateral amygdala, which in turn modulates activation in other brain regions implicated in memory consolidation, including the hippocampus and sensory cortices (Cahill and McGaugh, 1998; McGaugh et al., 2002; Roozendaal and McGaugh, 2011). Yet other human studies looking at emotionally enhanced perceptual activations under attentionally impoverished conditions have found a slightly different pattern of findings, with prefrontal mediation of the relation between the amygdala and visual cortex (Lim et al., 2009). Future research can use such approaches as dynamic causal modeling to investigate the directionality of amygdala influences on perceptual and mnemonic vividness.

It is important to highlight that our previously reported behavioral findings suggested that enhanced perceptual vividness contributes to, but does not entirely account for, the heightened salience of emotional memories (Todd et al., 2012). This is consistent with the current finding that the hippocampal and parahippocampal regions specifically implicated in memory formation and its emotional enhancement (Kensinger et al., 2007b; Talmi et al., 2007; Murty et al., 2010) were modulated by mnemonic – but not perceptual – vividness. Research in non-human animals (McGaugh et al., 2002) and humans (Cahill and Alkire, 2003; Anderson et al., 2006a) also indicates that arousal induced *after* encoding influences memory consolidation to alter memory retention, which by definition cannot be explained by vivid perceptual encoding. Phasic arousal related to perceptual vividness may interact with more tonic arousal extending beyond initial encoding to alter memory consolidation (Cahill and Alkire, 2003), mutually enhancing later memory. Thus, the influence of affective salience at encoding likely occurs at multiple timescales, which combine to endow emotional experiences and their associated memories with a uniquely vivid subjective character. Future research can delineate how individual differences in effects of arousal on perceptual vividness and post-encoding processes may be linked to normative differences in capacity for emotional memory as well as the prevalence of intrusively vivid memories following trauma (Nashiro and Mather, 2011; Todd et al., 2011).

## Conflict of Interest Statement

The authors declare that the research was conducted in the absence of any commercial or financial relationships that could be construed as a potential conflict of interest.
